# Global O-GlcNAcylation changes impact desmin phosphorylation and its partition toward cytoskeleton in C2C12 skeletal muscle cells differentiated into myotubes

**DOI:** 10.1038/s41598-022-14033-z

**Published:** 2022-06-14

**Authors:** Charlotte Claeyssen, Bruno Bastide, Caroline Cieniewski-Bernard

**Affiliations:** Univ. Lille, Univ. Artois, Univ. Littoral Côte d’Opale, ULR 7369 - URePSSS - Unité de Recherche Pluridisciplinaire Sport Santé Société, F-59000 Lille, France

**Keywords:** Intermediate filaments, Glycosylation, Phosphorylation

## Abstract

Desmin is the guardian of striated muscle integrity, permitting the maintenance of muscle shape and the efficiency of contractile activity. It is also a key mediator of cell homeostasis and survival. To ensure the fine regulation of skeletal muscle processes, desmin is regulated by post-translational modifications (PTMs). It is more precisely phosphorylated by several kinases connecting desmin to intracellular processes. Desmin is also modified by O-GlcNAcylation, an atypical glycosylation. However, the functional consequence of O-GlcNAcylation on desmin is still unknown, nor its impact on desmin phosphorylation. In a model of C2C12 myotubes, we modulated the global O-GlcNAcylation level, and we determined whether the expression, the PTMs and the partition of desmin toward insoluble material or cytoskeleton were impacted or not. We have demonstrated in the herein paper that O-GlcNAcylation variations led to changes in desmin behaviour. In particular, our data clearly showed that O-GlcNAcylation increase led to a decrease of phosphorylation level on desmin that seems to involve CamKII correlated to a decrease of its partition toward cytoskeleton. Our data showed that phosphorylation/O-GlcNAcylation interplay is highly complex on desmin, supporting that a PTMs signature could occur on desmin to finely regulate its partition (i.e*.* distribution) with a spatio-temporal regulation.

## Introduction

Intermediate filaments (IFs) are a major and elaborated cytoskeletal network that is at least ten-fold more abundant than microfilaments or microtubules^1^. The major function of IFs is the maintenance of cell shape and architecture, conferring resistance of cells towards mechanical stresses. They also contribute to the organization of cells because of their interaction with organelles. Indeed, IFs interact with mitochondria and modulate energy fluxes^2,3^. Moreover, they contribute to the organization of cells into tissues as IFs interact with adhesive structures such as focal adhesions or desmosomes^1^. Initially considered as static structures, it is nowadays well admitted that IFs are on the contrary highly dynamic and able to respond rapidly to cellular demands. Strong regulators of IFs dynamics are post-translational modifications (PTMs), in particular phosphorylation and O-*N*-acetyl-β-d-glucosaminylation (O-GlcNAcylation) that modulate the IFs dynamics and interactions in response to signalling pathways^4–7^. Beyond their mechanical role, IFs are a nodal point within cells because of their interaction with a growing number of proteins, in particular proteins of signalling pathways. As a consequence, IFs are both targets and active contributors of intracellular signalling. As modifiers and organizers of signalling, IFs contribute to a dynamic cell behaviour (development, proliferation, migration, apoptosis…) and in larger extent to tissue homeostasis (regeneration, wound healing…)^4,8–11^.

Among the constitutive proteins of IFs, desmin is the major protein of type III IFs in striated muscles as it represents 2% and 0.35% of total proteins in heart and skeletal muscles, respectively^12^. Desmin interactome is vast^13,14^. This protein forms a continuous transcytoplasmic network around Z-discs through specific interactions with a plethora of structural proteins, permitting the maintenance of lateral alignment of myofibrils, the cytoskeletal lattice of muscle and the anchoring of mitochondria and nuclei^13,15,16^. Over the preservation of muscle integrity and the involvement in contractile activity, desmin is also a key mediator of cellular homeostasis and survival because of its implication in several cellular processes such as differentiation, apoptosis, intracellular signalisation, mechanotransduction, vesicle trafficking, organelle biogenesis and/or positioning, calcium homeostasis, protein homeostasis and the regulation of metabolism and gene expression^12–14,17^. Desmin bears several PTMs, in particular phosphorylation by several kinases that connect desmin to different cellular processes such as cell division, myoblast differentiation or muscle contraction^18^. Hence, desmin is strongly associated to the aetiology of several muscle pathologies such as dilated cardiomyopathy, hypertrophic cardiomyopathy, restrictive cardiomyopathy, atypical cardiomyopathy, heart failure, sleep apnea, asthma, respiratory insufficiency, dysphagia, myofibrillar myopathies or muscle wasting^18–21^. Desmin-related myopathy and cardiomyopathy (i.e*.* desminopathies) could result from mutation in the desmin gene, cleavage of desmin, and/or changes on post-translational modifications, leading to an altered desmin network and the formation of toxic desmin aggregates^14,22–25^. As a consequence, desmin function is lost, leading to improper mitochondrial function, loss of cell adhesion and cell–cell communication, fibrosis and inflammation^14^. Importantly, these toxic aggregates are seeding-competent amyloid aggregates that persist in muscle fibers^26,27^, and these preamyloid and amyloid oligomers formation and deposition result from detrimental phosphorylation in heart failure^28,29^.

As mentioned previously, desmin is highly post-translationally modified, especially through phosphorylation^18^. However, desmin is not only phosphorylated but also modified by an atypical glycosylation: the *O*-*N*-acetyl-β-d-glucosaminylation (O-GlcNAcylation)^30^. The O-GlcNAcylation is an atypical glycosylation corresponding to the transfer of a unique monosaccharide, the *N*-acetyl-d-glucosamine, on serine or threonine hydroxyl group of a protein; O-GlcNAcylation is highly dynamic and reversible, presenting a dynamic interplay with phosphorylation^31^. The O-GlcNAcylation is highly abundant on cytoskeletal proteins; in particular, keratin 8 and 18 which are involved in the development and differentiation of various tissues are O-GlcNAcylated. Interestingly, while phosphorylation regulated protein interaction, ubiquitination and filament organization of keratins, O-GlcNAcylation was demonstrated to modulate the phosphorylation level of IFs^32,33^ and increase keratins solubility^5,34^, ubiquitination and proteasomal degradation of IFs proteins^35^. Moreover, O-GlcNAcylation was linked to the modulation of IFs, in particular vimentin^6^ and keratin 8/18 filaments^5,36^, with a link between O-GlcNAcylation and cell survival in stress condition^34^. In skeletal muscle, O-GlcNAcylation extensively modifies other nucleocytoplasmic and mitochondrial proteins and also myofibrillar proteins^37^. We have previously demonstrated that motor and regulatory proteins are O-GlcNAcylated modulating so the contractile properties of skeletal muscle such as calcium activation parameters^30,38–41^. Supporting a key role of this atypical glycosylation, the alteration in the number or the linkage of O-GlcNAc moieties is closely associated to the physiopathology of several diseases and in particular neuromuscular pathologies (polymyositis, dermatomyositis, sporadic inclusion body myositis, muscular dystrophies, neurogenic muscular atrophy, rhabdomyolysis, and distal myopathy with rimmed vacuoles)^42^. Interestingly, some of the O-GlcNAc sites correspond to mutated sites closely associated to the development of muscle pathologies such as Laing myopathy^43^ or desminopathies^44^. The O-GlcNAc modification was also demonstrated to be involved in skeletal muscle atrophy^39,45,46^. Moreover, recent data from our lab underlined the crucial role of O-GlcNAcylation in the modulation of contractile activity, in the structuration of sarcomeric cytoskeleton and in the modulation of protein–protein interactions (for review, see^37^). Indeed, we have recently demonstrated that the two enzymes responsible of the O-GlcNAcylation process are located in the nodal Z-line^39^, and that several myofibrillar proteins are O-GlcNAc-modified, such as αB-crystallin, α-actinin or desmin among others^30,38^. We have also demonstrated in a cellular model of C2C12 skeletal muscle cells differentiated into myotubes that O-GlcNAcylation could be involved in the organization and reorganization of sarcomere. Importantly, we showed that sarcomere reorganization was linked to a modulation of several multiprotein complexes and some of them include key structural proteins, in particular desmin and its molecular chaperone, the αB-crystallin^47^. To support this role of O-GlcNAcylation in the modulation of desmin interactome (in particular the interaction with αB-crystallin), O-GlcNAc sites modifying both proteins are located into interaction domains^44^, and our data also suggest that their interaction could be impacted by O-GlcNAcylation changes^47^.

However, the precise impact of O-GlcNAcylation on desmin, the guardian of striated muscle integrity, remains poorly understood. The major objective of our study was to reveal the role of O-GlcNAcylation on desmin features. For this purpose, we increased or decreased the global O-GlcNAcylation level on C2C12 myotubes using pharmacological molecules inhibiting OGA or OGT (the two enzymes involved in O-GlcNAcylation and de-O-GlcNAcylation process) respectively, and investigated whether the global O-GlcNAcylation level should impact the desmin behaviour or not. We focused particularly on its expression, its partition toward cytoskeleton or insoluble fraction, and the variations of phosphorylation and O-GlcNAcylation on desmin.

## Materials and methods

### C2C12 cells culture

#### Myoblasts proliferation and differentiation

The C2C12 mouse myoblasts (ATCC: American Type Culture Collection, Manassas, VA) were cultured in proliferation medium [PM: Dulbecco’s Modified Eagle’s Medium (DMEM, Gibco); 10% foetal calf serum (Gibco); 1% antibiotics/antimycotics (Sigma)]. Cells were put in plate of 8.8 cm^2^ at a density of 2 × 10^5^ cells/ml in PM. When reaching 80–90% confluence, PM was changed toward differentiation medium [DM: DMEM (Gibco); 2% heat inactivated horse serum (Gibco); 1% antibiotics/antimycotics (Sigma)]. This medium change corresponds to day 0 of differentiation. Cells were cultured into an incubator at 37 °C, 5% CO_2_ and saturated humidity, and culture media were changed every 2 days.

#### Pharmacological treatments

After 5 days of differentiation, C2C12 myotubes were treated by pharmacological molecules applied overnight. We used 5 µM of Ac_4_-5S-GlcNAc (peracetyl-5-thio-GlcNAc; generous gift from Pr D.Vocadlo, Simon Fraser University, Burnaby, Canada)^48^ or 0.5 µM of Thiamet-G (thiazoline amino-ethyl gluco-configured)^49^ resolubilized in DMSO (dimethyl sulfoxide) and diluted in DM, to inhibit OGT and OGA respectively. Control myotubes were cultured in DM with DMSO as vehicle but without OGT or OGA inhibitors. To increase or decrease CamKII activity, we applied overnight 3 mM of caffeine^50^ (Thermofisher) or a co-treatment with 3 mM of caffeine added to 20 µM of KN-93^51^ resolubilized in water.

### Proteins extraction and fractionation

**Figure 1:**
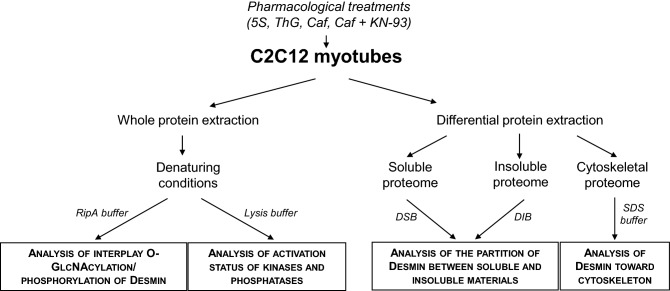
Recapitulative scheme of the different protein extraction protocols. This scheme indicated the protein subcellular fractions obtained, the corresponding buffers used and the downstream analyses for each fraction.

Cells were rinsed three times with ice-cold PBS (Phosphate Buffer Saline). Proteins were extracted by cells scraping and lysis with 150 μl of extraction buffer adapted to downstream analyses according to specific protocols defined hereinafter. All extraction buffers contained anti-proteases [Complete EDTA-Free (Roche Diagnostics)], anti-phosphatases [Phos-Stop (Roche Diagnotics)] and 50 μM of PUGNAc (*O*-(2-acetamido-2-deoxy-d-glucopyrano-silidene)-amino-*N*-phenyl-carbamate; Sigma), an OGA inhibitor. A recapitulative scheme of all extraction protocols is presented in Fig. 1.

#### Whole cellular extract

RIPA buffer (10 mM Tris/HCl, pH 7.4; 150 mM NaCl; 1 mM EDTA; 1% Triton X-100; 0.5% sodium deoxycholate; 0.1% SDS) was used to get whole extract. Protein content was estimated by Bradford assay.

#### Cellular extract for kinases and phosphatases assays

For kinase assays, cells were lysed in lysis buffer (20 mM MOPS; 5 mM EGTA; 1% NP40; 1 mM DTT) according to manufacturer’s protocol, and after centrifugation at 13,000 rpm for 10 min at 4 °C, supernatant protein content was determined with BCA assay. For phosphatase assay, RIPA buffer without SDS and phosphatases inhibitors was used to get whole extract. Protein content was estimated by Bradford assay.

#### Cytoskeletal fraction

Cells were lysed with Cell Lysis Buffer (50 mM PIPES, pH 7.4; 50 mM NaCl; 5% glycerol; 0.1% Triton X-100; 0.1% Tween-20) 1.5 min on ice^52^. The solution was discarded. After rinsing with 300 μl Tris/HCl buffer (50 mM Tris HCl pH 7.5), 100 μl of nuclease Buffer (50 mM Tris/HCl, pH 7.4; 10 mM MgCl_2_; 2 mM CaCl_2_) supplemented with 10 U/ml of benzonase nuclease were applied for 10 min; the solution was discarded too. Cytoskeletal proteins that remained fixed on the plate were rinsed by Tris/HCl buffer and then solubilized by scrapping with 30 μl of Tris/HCl buffer supplemented by 1% SDS^52^. Protein content of SDS-resuspended extracts, containing cytoskeletal proteins was estimated by Lowry assay.

#### Insoluble and soluble fractions

Cells were lysed by Detergent Soluble Buffer (DSB: 50 mM Tris/HCl, pH 7.4; 150 mM NaCl; 1 mM EDTA; 1% Triton X-100) after 30 min on ice. Protein extract was centrifuged at 13,000 rpm for 10 min at 4 °C^53^. Supernatant corresponds to soluble fraction; pellet, corresponding to insoluble proteins fraction, was resolubilized by 100 μl of Detergent Insoluble Buffer (DIB: 50 mM Tris/HCl, pH 6.8; 2% SDS; 100 mM DTT; 10% glycerol)^53^. Protein content of DSB extracts was estimated by Bradford assay while those corresponding to DIB extract was estimated by Lowry assay.

#### Dephosphorylation process

Twenty micrograms of proteins were incubated in dephosphorylation buffer (50 mM Tris/HCl, pH 7.6; 100 mM NaCl; 1 mM DTT, 10 mM MgCl_2_; 1 mM MnCl_2_, added with anti-proteases [Complete EDTA-Free (Roche Diagnostics)], 10–20 U alkaline phosphatase (Sigma P6774) for 8 h at 37 °C. Then, proteins were denatured by boiling in Laemmli buffer.

### Immunoprecipitation

One hundred µg of proteins in RIPA were incubated with protein G coupled on magnetic bead (Millipore) during 1 h at 4 °C for pre-clearing step. The non-retained sample was incubated with primary antibody overnight at 4 °C with gentle agitation, followed by 2 h incubation with magnetics beads (1:5). Beads were then washed sequentially using RIPA, RIPA + 0.5 M NaCl, RIPA + TNE (10 mM Tris/HCl, pH 7.4; 150 mM NaCl; 1 mM EDTA) (50:50, v/v) and lastly with TNE. Beads were finally resuspended in Laemmli buffer (62.5 mM Tris/HCl, pH 6.8; 10% glycerol; 2% SDS; 5% β-mercaptoethanol; 0.02% bromophenol blue) and boiled during 8 min at 95 °C. The soluble fraction corresponding to the immunoprecipitated proteins was analysed by SDS-PAGE and western-blot as described below.

### Electrophoretic methods

#### SDS-PAGE

Proteins samples were separated by SDS-PAGE on 7.5% Stain-free acrylamide or AnykD Mini-PROTEAN TGX Stain-free (SF) Precast Gels (Biorad). The electrophoretic separation was done at constant voltage (300 V) for about 20 min in a tank with migration buffer (190 mM Glycine; 25 mM Tris Base; 0.1% SDS). At the end of the electrophoretic separation, total proteomes were visualized after UV activation with ChemiDoc MP Imager (Imaging System) and ImageLab software (Biorad) by means of the Stain-Free technology. It is based on trihalo compound incorporation in the gel, which reacts with proteins and covalently binding to tryptophan residues, rendering them detectable after UV exposure. Proteins were then transferred on 0.2 μm nitrocellulose membrane using Trans-blot Turbo transfer system (Biorad) at 1.3 A, up to 25 V/gel during 10 min. The quality of transfer was checked by imaging of the SF profile with ChemiDoc MP Imager.

#### Phos-tag-PAGE

Proteins samples were resolved on Phos-tag-PAGE gel^54,55^, composed of stacking gel (4% acrylamide/bisacrylamide [29:1]; 125 mM Tris/HCl, pH 6.8; 0.01% SDS; 0.06% APS 10%; 0.034% TEMED) and resolving gel (7.5% acrylamide/bisacrylamide [29:1]; 375 mM Tris/HCl pH 8.8; 0.01% SDS; 10 mM MnCl_2_; 20 μM Phos-tag™ acrylamide [AAL-107, NARD Institute]; 0.5% APS 10%; 0.1% TEMED). Electrophoretic migration was performed using migration buffer as described above at constant amperage (25 mA/gel) for about 110 min. After migration, Phos-tag gels were incubated twice for 7 min in transfer buffer (20% ethanol; 20% Transfer buffer 5 × [Trans-blot turbo, Biorad]) supplemented with 1 mM EDTA, and then two times in transfer buffer without EDTA. Finally, proteins were transferred on 0.2 μm PVDF membrane with Trans-blot Turbo transfer system as described above. The quality of transfer was checked by imaging using the ChemiDoc MP Imager after membrane staining with red Ponceau (Thermofisher).

#### Western-blot

Membranes were blocked with 5% BSA for RL-2, CamKII and P-CamKII antibodies or non-fat dry milk for desmin, PAK1 and P-PAK1/2 antibodies in TBS-T (Tris Buffer Saline-Tween: 15 mM Tris/HCl, pH 7.6; 140 mM NaCl; 0.05% Tween-20). Membranes were incubated with primary antibody as follow: RL-2 (MA1-072, ThermoFisher) at 1/2500^e^; Desmin (Abcam ab6322) at 1/50000^e^; CamKII (Cell Signaling #4436) at 1/1000^e^; P-CamKII (Thr286) (Cell Signaling #12716) at 1/1000^e^; PAK1 (Cell Signaling #2602) at 1/1000^e^; P-PAK1/2 (Ser144/Ser141) (Cell Signaling #2606) at 1/1000^e^ in blocking solution overnight at 4 °C with gentle agitation. After three washes of 10 min in TBS-T, membranes were incubated in blocking solution containing secondary antibodies (HRP-linked IgG, Cell Signaling #7074 or #7076) for 1 h at RT before five washes of 10 min each in TBS-T. Signals were detected by chemiluminescence using ECL Clarity (Biorad) and ChemiDoc MP. Images were acquired and analysed using Image Lab^®^ software.

### Kinases and phosphatases activity assay

#### PKA and PKC activities assay

The activity of PKA and PKC were measured using PKA and PKC activity assay kits (Abcam 139435 and 139437, respectively) according to the manufacturer’s protocol. Once proteins extracted according to the protocol defined for kinases assays, 1 µg of protein extract was used for ELISA assay based on a specific phospho-peptide substrate which was phosphorylated by active PKA or PKC. The phosphorylation level of peptide substrate was determined by absorbance measurement at 450 nm on SpectraMax reader (Molecular devices).

#### In-gel phosphatase assay

The phosphatases activities were in-gel assayed using the fluorogenic substrate MUP (4-methyl-umbelliferyl phosphate), MUP being converted to fluorescent products once hydrolysed by phosphatase(s)^56,57^. Native-PAGE was performed with 75 µg proteins extracted using the same procedure than SDS-PAGE except that SDS was removed from RIPA buffer, Laemmli buffer, migration buffer, 10% acrylamide/bisacrylamide [37.5:1] separating gel and 4% stacking gel. The electrophoretic separation was done under constant amperage (20 mA/gel) at 4 °C to prevent loss of phosphatase activity for about 110 min. Once the electrophoretic separation was achieved, the native gels were incubated in reaction mixture (50 mM Tris/HCl, pH 8; 0.1 mM EGTA; 0.01% Tween 20; 20 mM β-mercaptoethanol; 20 mM MnCl_2_; 0.5 mM MUP at 10 mM in DMSO) for 15 min at 37 °C with manual shaking every minute as previously described^56,57^. Fluorescent bands corresponding to phosphatases activity were detected after UV exposition (365 nm) using ChemiDoc MP.

### Statistical analysis

The quantification of signal intensities was carried out following normalization by total protein level on Stain-Free images or by internal standards. Means of each group were related to the corresponding control mean having the value of 1. The significance of intergroup differences was determined by Kruskal–Wallis test (GraphPad Prism software). Experiments were done on 5 dishes per culture, on 3 independent cultures (n = 15, N = 3) and samples were distributed on several gels performed in parallel. Results were presented as mean ± SEM and outlier values were removed when existing. Significance levels were indicated by *p < 0.05, **p < 0.01 or ***p < 0.001.

## Results

### Modulation of O-GlcNAcylation global level

To better understand the phosphorylation/O-GlcNAcylation interplay on desmin, the global O-GlcNAcylation level was modified in 5-days differentiated C2C12 myotubes using pharmacological molecules. Thiamet G^48^ and Ac_4_-5S-GlcNAc^49^, OGA and OGT inhibitors, were used to increase and decrease the global O-GlcNAcylation level. For each treatment, we determined the differentiation status (fusion index) and cell viability (colorimetric MTT assay) which were not altered whatever the treatment applied (Supplemented Fig. 1).

**Figure 2:**
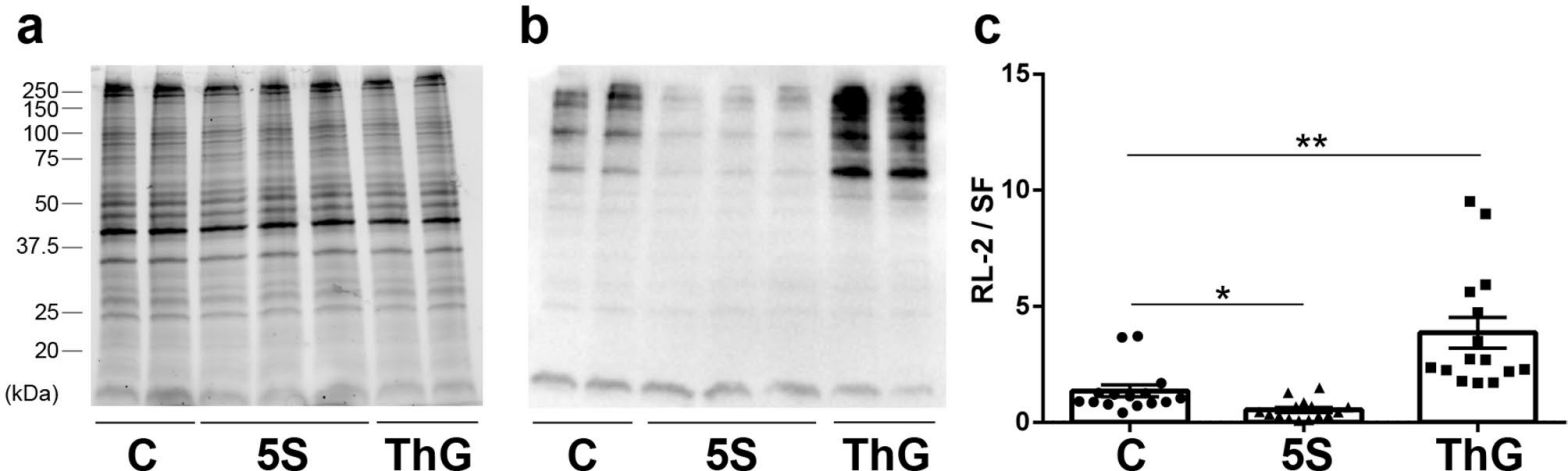
Quantification of global O-GlcNAcylation level in C2C12 myotubes following Ac_4_-5S-GlcNAc (5S, ▲) or Thiamet G (ThG, ■) treatment. Twenty µg of proteins sample extracted with RIPA buffer were resolved on 7.5% acrylamide Stain-free gels and analysed by western blot. (**a**) Whole protein profiles detected using Stain-Free technology. (**b**) O-GlcNAcylation pattern revealed with RL-2 antibody. (**c**) Quantification of western blot signals, normalized to Stain-Free profile. Data were expressed as mean ± SEM and compared with the control condition. *p < 0.05, **p < 0.01 significantly different from control (n = 15, N = 3 independent cultures). Uncropped images of gel and blot are presented in Supplemental data.

To quantify changes of the global O-GlcNAcylation level, we performed western blot using RL-2 antibody which recognizes O-GlcNAc moieties; whole protein profiles (Stain-Free) and RL-2 profiles were presented on Fig. 2a,b, respectively. The quantification of the variation of global O-GlcNAcylation level was presented on Fig. 2c. As shown on Fig. 2, Ac_4_-5S-GlcNAc treatment led to a significant decrease of O-GlcNAcylation level (0.54 ± 0.11, p < 0.05) while Thiamet G treatment significantly increased the O-GlcNAcylation level (3.87 ± 0.66, p < 0.01) compared to control cells (1.00 ± 0.26).

### Modulation of desmin partition between soluble and insoluble fractions and towards cytoskeleton

**Figure 3:**
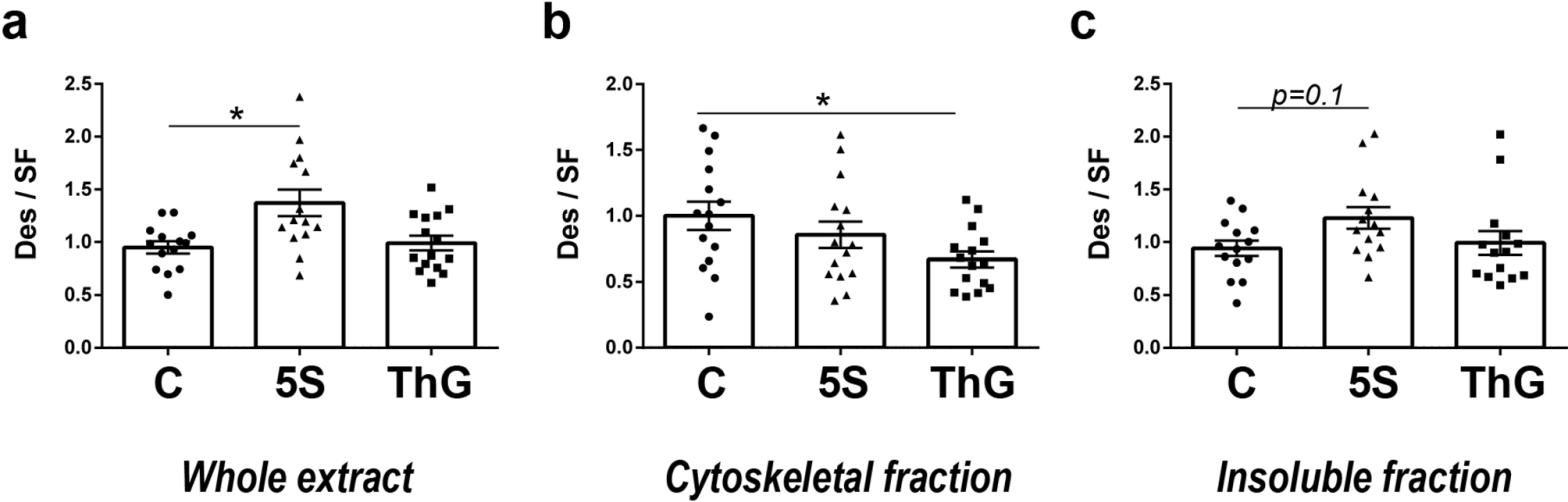
Quantification of desmin partition toward cytoskeleton or insoluble protein material. Twenty µg of proteins sample extracts (RIPA for desmin protein expression, and SDS-resolubilized extracts or DIB for partition toward cytoskeleton or insoluble material, respectively) were resolved on AnykD Stain-free gels and analysed by western blot for desmin detection. (**a**) Desmin protein level in whole proteome. (**b**,**c**) Changes in desmin partition towards cytoskeleton (**b**) or insoluble protein material (**c**) from untreated (C, ●), Ac_4_-5S-GlcNAc- and Thiamet G-treated myotubes (5S, ▲ and ThG, ■ respectively). Data were expressed as mean ± SEM and compared with the control condition. **p < 0.01: significantly different from control (n = 15; N = 3 independent cultures).

The desmin protein level was quantified in the whole proteome (Fig. 3a). While the Thiamet G treatment didn’t impact the desmin protein level compared with control (0.99 ± 0.07), its protein level increased significantly in the whole extract following a global O-GlcNAcylation decreased resulting from Ac_4_-5S-GlcNAc (1.37 ± 0.13, p < 0.05).

Myotubes proteins were extracted according to two differential extraction protocols permitting to access to soluble and insoluble protein fractions or to cytoskeleton, as described in Fig. 1. The different proteins patterns and the validation of differential extraction were presented on Supplemented Fig. 2. The partition of desmin investigated by western blot performed on insoluble fraction and on cytoskeletal proteins was modified consecutively to global O-GlcNAcylation changes. Figure 3b showed that Thiamet G treatment significantly decreased the partition of desmin toward cytoskeleton (0.67 ± 0.06, p < 0.05) compared to control (1.00 ± 0.11) while this treatment didn’t modify desmin partition into the insoluble fraction (Fig. 3c). The Ac_4_-5S-GlcNAc treatment slightly increased desmin in insoluble fraction (1.23 ± 0.10, p = 0.1) compared to control (0.94 ± 0.07) (Fig. 3c); however, it didn’t cause any change of desmin partition toward cytoskeleton (Fig. 3b).

### Phosphorylation/O-GlcNAcylation interplay of desmin

**Figure 4:**
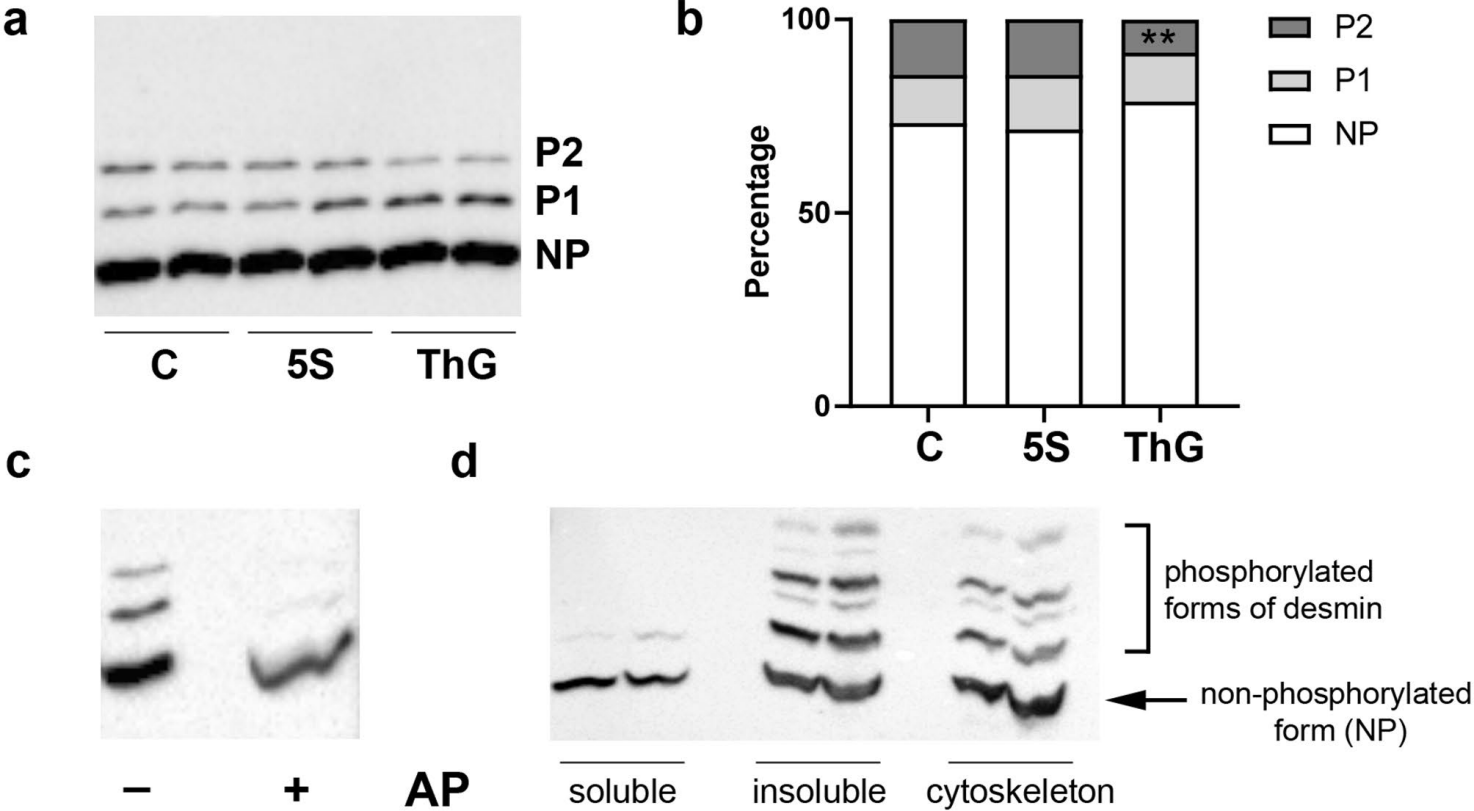
Quantification of the phosphorylated forms of desmin in C2C12 differentiated myotubes consecutively to global O-GlcNAcylation variations. Twenty µg of whole proteins samples (RIPA extracts) or protein samples resulting from differential extractions (DSB: soluble; DIB: insoluble; and SDS-resolubilized: cytoskeleton) were separated using Phos-tag-PAGE, and desmin was detected using western blot. (**a**,**b**) Quantification of desmin phosphorylation changes in whole protein extract of untreated (C), Ac_4_-5S-GlcNAc- and Thiamet G-treated myotubes (5S, and ThG respectively). The phosphorylated forms of desmin were separated according to the number of phosphate groups (P1: first retarded form of desmin; P2: second retarded form of desmin) comparing with the non-phosphorylated form of desmin (NP); the representative pattern in untreated and treated myotubes was presented on (**a**) panel. The signals were quantified and expressed as the relative percentage of each form (NP, P1 and P2 forms, comparing with the sum of all forms (NP + P1 + P2, equal to 100%, for each condition). (**c**) A control sample was dephosphorylated using alkaline phosphatase (AP) prior to Phos-tag-PAGE and detection of desmin was carried out through western blot. (**d**) Protein samples resulting from differential extractions (DSB: soluble; DIB: insoluble; and SDS-resolubilized: cytoskeleton) were separated using Phos-tag-PAGE, and detection of desmin was carried out through western blot. **p < 0.01: significantly different from control (n = 15; N = 3 independent cultures). Uncropped images of blots are presented in Supplemental data.

The variation of phosphorylation level was performed using a gel electrophoresis approach based on Phos-tag-PAGE^54,55^, permitting the retardation of the different phosphorylated forms of a protein compared to the non-phosphorylated one; the protein of interest was then detected by western-blot. As indicated on Fig. 4a, the non-retarded band (assigned as NP band) corresponded to the non-phosphorylated form of desmin in whole protein extract. The retarded bands were assigned to P1 and P2, corresponding to the first and the more retarded band, respectively. In order to validate this retardation as phosphorylated forms of desmin comparing with the non-phosphorylated one, a control sample was dephosphorylated using alkaline phosphatase prior to Phos-tag-PAGE (Fig. 4c).

All signals were quantified, and each band was expressed as relative percentage compared with the sum of each signal (NP, P1 and P2) that corresponds to whole desmin protein level. Data were represented on Fig. 4b. We showed that the increase of global O-GlcNAcylation level through the Thiamet G treatment of myotubes led to a change in its global level of phosphorylation (Fig. 4b) which was characterized by a significant decrease of the most retarded form (band P2) (8.58% versus 14.36% for control, p < 0.01) (Fig. 4b). We didn’t quantify any change for the non-phosphorylated form (NP), nor any difference in the phosphorylation pattern in Ac_4_-5S-GlcNAc myotubes.

We also studied the protein pattern of the protein extracts resulting from the differential extraction protocols using Phos-tag-PAGE. As shown on Fig. 4d, several phosphorylated forms of desmin could be detected on cytoskeletal or on insoluble fractions. In contrast, a very slight signal corresponding to phosphorylated desmin was detected on soluble fraction, supporting that phosphorylation impacts the partition of desmin towards insoluble fraction or towards cytoskeleton. In addition, we observed additional bands in the insoluble and cytoskeleton fractions that were not detected in the whole extract. This suggests that phosphorylation status on desmin partition toward specific protein fraction such as cytoskeleton plays a crucial role.

### Modulation of desmin O-GlcNAcylation

**Figure 5:**
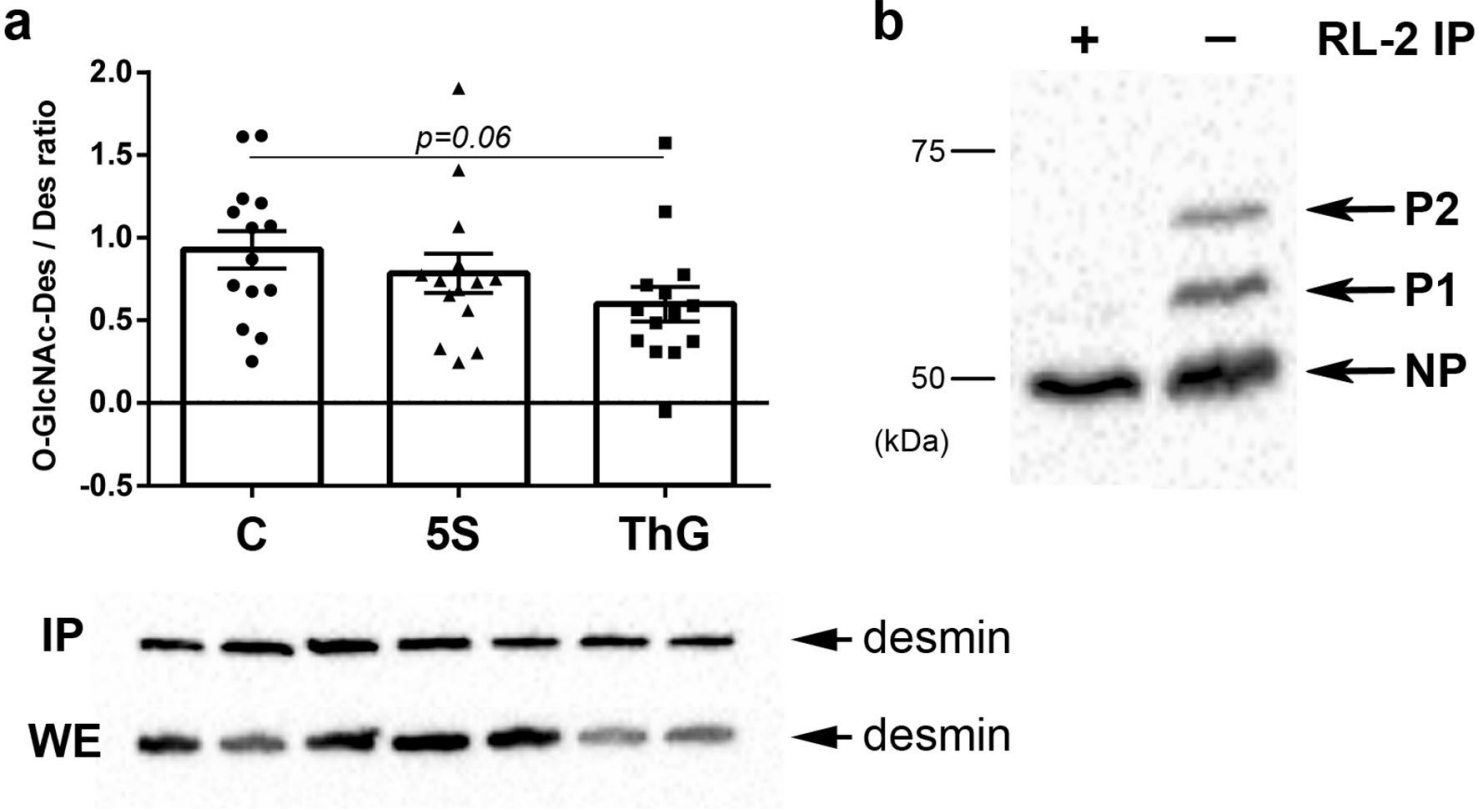
Quantification of desmin O-GlcNAcylation and its interplay with phosphorylation. Changes of desmin O-GlcNAcylation level were determined in whole extract by western blot analysis performed after immunoprecipitation of O-GlcNAcylated proteins from untreated myotubes (C, ●), and Ac_4_-5S-GlcNAc- and Thiamet G-treated myotubes (5S, ▲ and ThG, ■ respectively). (**a**) Signals were quantified and expressed as ratio of glycosylated protein/protein level (mean ± SEM), and compared to the control condition. The representative signals for whole extract (WE) and the immunoprecipitated proteins (IP) were shown under the histogram. (**b**) Desmin was detected using western blot following Phos-tag-PAGE after immunoprecipitation of O-GlcNAcylated proteins (left lane) comparing with the whole protein extract from untreated C2C12 myotubes (right lane). NP corresponded to the non-phosphorylated desmin, and P1 and P2 the phosphorylated forms of desmin. The tendency of difference comparing with control is indicated on histogram (n = 15; N = 3 independent cultures). Uncropped images of blots are presented in Supplemental data.

We performed western-blot directed against desmin following immunoprecipitation of O-GlcNAcylated proteins using RL-2 antibody as previously described in previous papers of the lab^39,47^. This approach allowed to determine that desmin O-GlcNAcylation slightly decreased in whole extract after Thiamet G treatment (0.60 ± 0.11, p = 0.06) compared to control (0.93 ± 0.11) (Fig. 5a) while it didn’t change following the Ac_4_-5S-GlcNAc treatment.

To clarify the link between phosphorylation and O-GlcNAcylation of desmin, we immuno-precipitated O-GlcNAcylated proteins from whole protein extract of control C2C12 myotubes prior to Phos-tag-PAGE; desmin detection was carried out by western blot. As shown on Fig. 5b, our data indicated that the O-GlcNAcylated desmin was not phosphorylated as no retarded bands were observed after RL-2 immunoprecipitation (Fig. 5b, left lane) while we detected two retarded forms in the whole extract (Fig. 5b, right lane). These data suggest that the two post-translational modifications are mutually exclusive for desmin.

### Analysis without an a priori of phosphatases activity that could be involved in the variation of desmin phosphorylation

**Figure 6:**
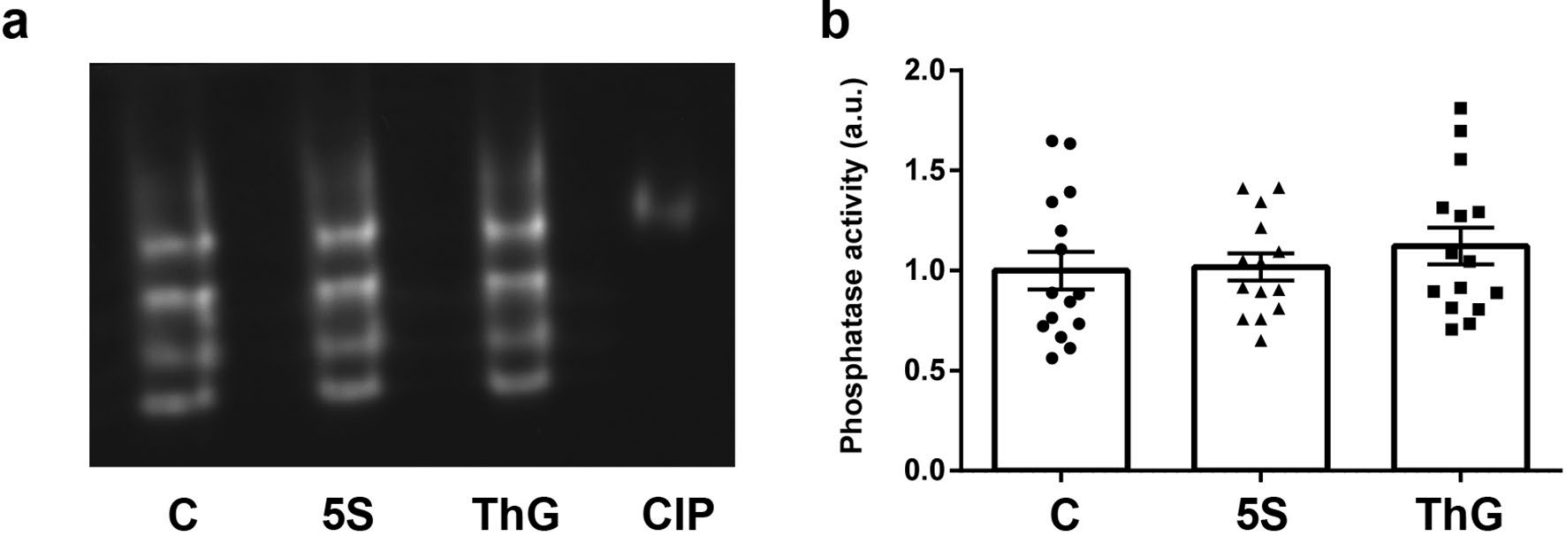
Quantification without an a priori of phosphatase activities involved in the desmin dephosphorylation. (**a**) In-gel detection of phosphatases activity after separation of 75 µg of proteins extracted from untreated myotubes (C, ●), and Ac_4_-5S-GlcNAc- and Thiamet G-treated myotubes (5S, ▲ and ThG, ■ respectively) using 10% Native-PAGE; MUP was used as phosphatase substrate, leading to fluorescence emission once hydrolysed by phosphatases. The Calf Intestine Phosphatase (CIP, 1 mU) was used as positive control. (**b**) Quantification of total phosphatases activities. Resulting signals were quantified, expressed as mean ± SEM and compared with the control condition (n = 15; N = 3 independent cultures). Uncropped image of gel is presented in Supplemental data.

To go further in understanding the complex phosphorylation/O-GlcNAcylation interplay, we analysed the activity of phosphatases that could be involved in desmin dephosphorylation. Indeed, a modulation of the phosphorylation of a given protein could result from a modulation of kinase(s) activity but also phosphatase(s) activity. To identify without a priori the phosphatases presenting a change in their activity, we performed an in-gel protein phosphatase assay using 4-methyl-umbelliferyl phosphate substrate (MUP) (Fig. 6a)^57^. This approach allowed us to determine that there was no changes of global phosphatases activity whatever the treatment applied (Fig. 6b); in the same way, we didn’t observe any change for each separated band (Supplemented Fig. 3).

### Identification of kinase(s) involved in the variation of desmin phosphorylation

We have demonstrated in the herein paper that increased O-GlcNAcylation level led to a significant decrease of desmin phosphorylation without alteration of phosphatases activity. To go further in the fine understanding of the interplay between phosphorylation and O-GlcNAcylation, we attempted to determine the kinase(s) involved in this decrease of phosphorylation level. We quantified PKA and PKC activities using ELISA approach and we determined the activation status of CamKII and PAK using the phosphorylation level of these enzymes, which is known to lead to their activation; the representative signals for CamKII, P-CamKII, PAK1 and P-PAK1/2 were presented in Supplemented Fig. 4.

**Figure 7:**
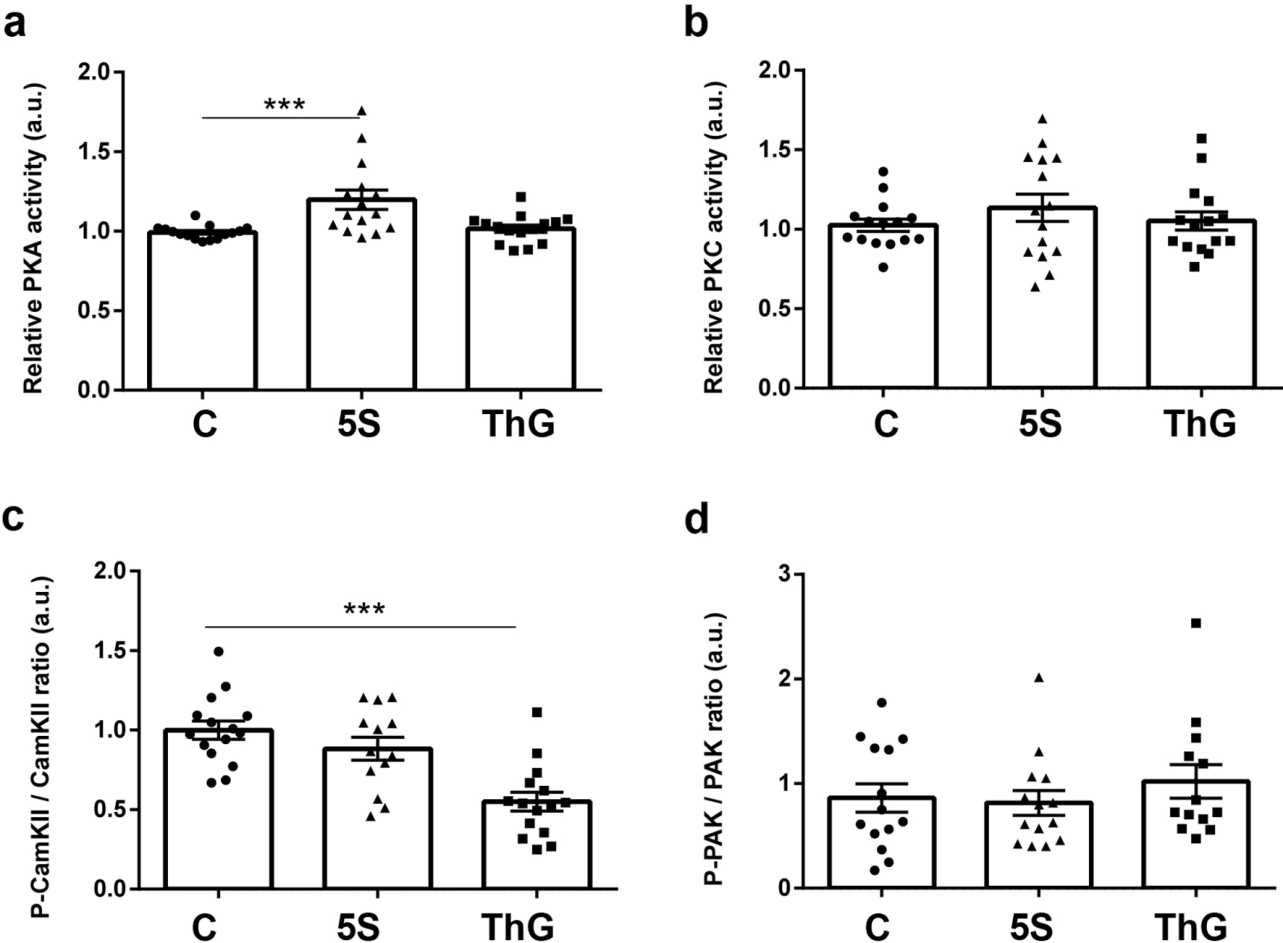
Quantification of the activity/activation status of the major kinases involved in the desmin phosphorylation. The PKA and PKC activities were assayed by ELISA, while activation status of CamKII and PAK was investigated by western blot. **(a**,**b**) Quantification of PKA (**a**) and PKC (**b**) activities. (**c**,**d**) Quantification of the phosphorylation status of CamKII (**c**) and PAK (**d**), the phosphorylation level translating their activation status. The signals intensity of phospho-CamKII and phospho-PAK were normalized according to the expression level of CamKII and PAK, and expressed as phosphorylated signal/whole signal ratio. Data were expressed as mean ± SEM and compared with the control condition. ***p < 0.001: significantly different from control. (n = 15; N = 3 independent cultures).

**Figure 8:**
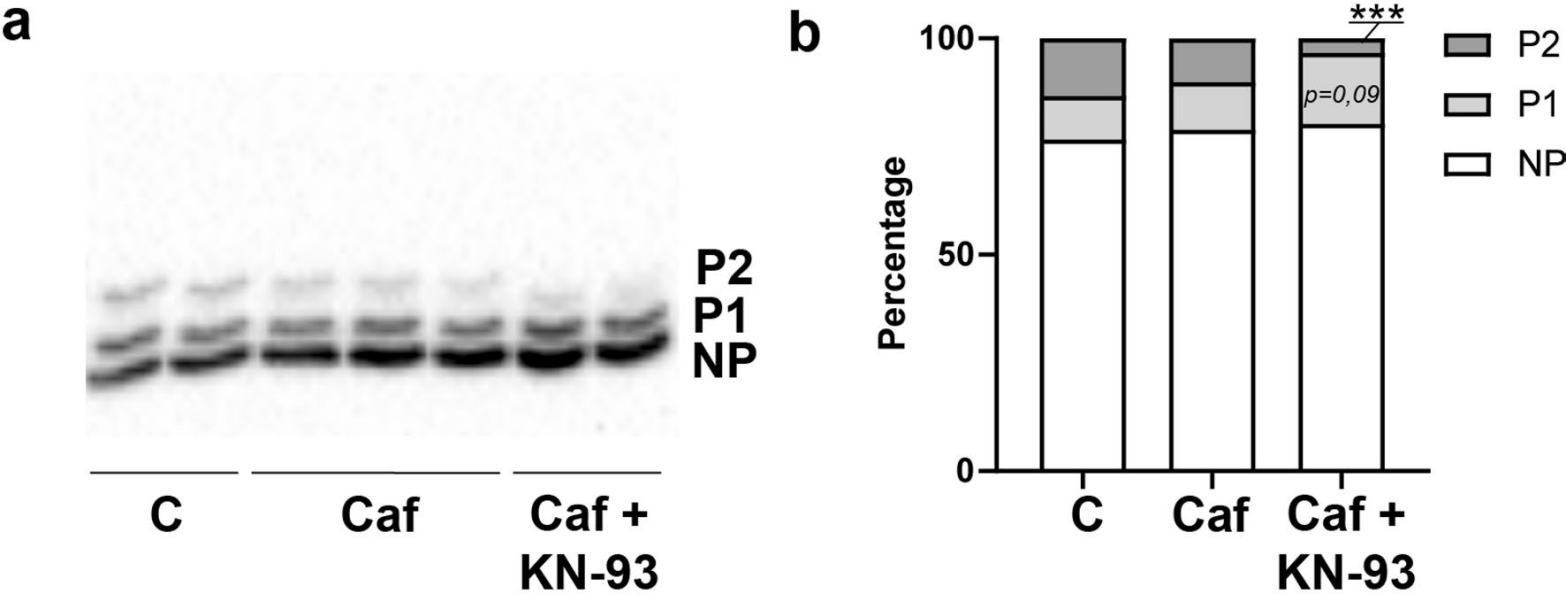
Quantification of the phosphorylated forms of desmin in C2C12 differentiated myotubes consecutively to co-treatment caffeine and KN-93, CamKII activator and inhibitor, respectively. Twenty µg of the whole proteins samples were separated using Phos-tag-PAGE, and desmin was detected using western blot. (**a**) Visualization of phosphorylated pattern of desmin in untreated (C), caffeine (Caf) and caffeine + KN-93 (Caf + KN-93) treated myotubes. The phosphorylated forms of desmin were separated according to the number of phosphate groups (P1: first retarded form of desmin; P2: second retarded form of desmin) comparing with the non-phosphorylated form of desmin (NP). (**b**) The signals were quantified and expressed as the relative percentage of each form (NP, P1 and P2 forms, comparing with the sum of all forms (NP + P1 + P2, equal to 100%, for each condition). ***p < 0.001: significantly different from control (n = 15; N = 3 independent cultures). Uncropped image of blot is presented in Supplemental data.

As shown on Fig. 7a, the PKA activity increased consecutively to global decrease of O-GlcNAcylation level in Ac_4_-5S-GlcNAc-treated myotubes (1.20 ± 0.06, p < 0.001) compared to control (0.99 ± 0.01), while its activity was unmodified following the increase of global O-GlcNAcylation level in Thiamet-G-treated myotubes. The PKC activity was not modified whatever the variation of the global O-GlcNAcylation level (Fig. 7b). Interestingly, our data showed that the global increase of O-GlcNAcylation consecutively to Thiamet G treatment led to a significant decrease of CamKII phosphorylation (0.55 ± 0.06, p < 0.001) compared to control (1.00 ± 0.06), translating a decrease in its activation level (Fig. 7c). This data could explain the decrease of desmin phosphorylation we quantified in Thiamet G-treated myotubes. Lastly, we determined whether the activation status of PAK could be impacted by global O-GlcNAcylation level variation. However, we didn’t quantify any change of activation status of PAK (Fig. 7d). Interestingly, when CamKII activity was modified consecutively to co-treatment with caffeine (CamKII activator) and KN-93 (CamKII inhibitor), we observed a decrease in the P2 signal (3.36% versus 13.33% for control, p < 0.001) on Phos-Tag PAGE while the P1 signal slightly increased (16.44% versus 10.05% for control, p = 0.09) (Fig. 8). The non-phosphorylated (NP) signal remained unchanged. These data supported that CamKII could be involved in the regulation of the desmin phosphorylation level.

## Discussion

In the herein paper, we determined whether the O-GlcNAcylation level modulation could impact desmin behaviour in C2C12 myotubes. For this purpose, O-GlcNAcylation level was pharmacologically modulated with specific inhibitors of OGT and OGA (the Ac_4_-5S-GlcNAc^48^ and the Thiamet-G^49^, respectively). Our data support that O-GlcNAcylation impacts desmin protein level, its phosphorylation and O-GlcNAcylation as well as its subcellular distribution (see recapitulative Table 1). Interestingly, while we expected an opposite impact of the two inhibitors, we observed differential changes of desmin behaviour according to global O-GlcNAcylation variations. This suggests that targeted inhibition of OGT or OGA led to differential adaptive response of myotubes. Indeed, when global O-GlcNAcylation level decreased following Ac_4_-5S-GlcNAc treatment, we showed an increase of desmin protein level that was preferentially found into the insoluble fraction. In contrast, the desmin protein level remained unmodified when O-GlcNAcylation was increased following Thiamet G treatment; however, the partition of desmin toward cytoskeleton decreased, suggesting that O-GlcNAcylation could be involved in cytoskeleton remodelling.

**Table 1:** Recapitulative table of desmin behaviour in Ac_4_-5S-GlcNAc- and Thiamet G-treated myotubes, leading to a decrease and increase of global O-GlcNAcylation level, respectively. In this table the changes of desmin protein level and partition of desmin towards cytoskeleton and insoluble materials were represented. The variations of desmin O-GlcNAcylation and phosphorylation were also considered, as well as the kinases and phosphatases activities. Variations were compared with control myotubes. Significance levels were indicated by *p < 0.05, **p < 0.01, ***p < 0.001, or ~ for 0.05 < p < 0.1.

	Ac_4_-5S-GlcNAc-treated myotubes	Thiamet G-treated myotubes	Corresponding figure
Global O-GlcNAcylation level			Figure 2
Desmin protein level		=	Figure 3a
**Partition**			
*Cytoskeleton*	=		Figure 3b
*Insoluble*		=	Figure 3c
Desmin O-GlcNAcylation	=		Figure 5
Desmin phosphorylation	=		Figure 4
Phosphatases activity	=	=	Figure 6 + S3
**Kinases activity**			
*PKA*		=	Figure 7a
*PKC*	=	=	Figure 7b
*CamKII*	=		Figure 7c + S4a
*PAK*	=	=	Figure 7d + S4b

Another important finding is that global O-GlcNAcylation impacts desmin phosphorylation. Indeed, while the Ac_4_-5S-GlcNAc had no effect on desmin phosphorylation, the increase of global O-GlcNAcylation level through Thiamet G treatment led to a decrease of desmin phosphorylation. Phosphorylation/dephosphorylation is a central process that modulates the IFs dynamics and function. Per se, phosphorylation dictates the intrinsic properties of IFs such as filament organization, turnover, binding with associated proteins or solubility. It could particularly impact the redistribution between soluble pool and filamentous and insoluble material^58^. However, the phosphorylation process on desmin is highly complex, depending on several kinases that link desmin to several cellular processes^18^. In vitro phosphorylation of the desmin head domain is often described to contribute to IFs solubilisation^59^, but in contrast, phosphorylation, and more specifically hyperphosphorylation, is closely linked to the physiopathology of skeletal muscle diseases (in particular myofibrillar myopathies) or in heart disorders (such as heart failure), characterized by desmin aggregation^24,28,29,60–62^.

While the decrease of the phosphorylation level was correlated with a decrease of the partition of desmin toward cytoskeleton, we investigated whether desmin could be differentially phosphorylated in cytoskeletal or in soluble/insoluble fractions. We thus analysed the phosphorylated pattern of desmin in each of these fractions using Phos-tag-PAGE^39,54,55^; this approach was previously applied to analyse the pattern of phosphorylation of desmin in cardiomyocytes and heart tissue^24,28,62,63^. However, to our knowledge, this approach was applied for the first time in skeletal muscle cells considering cytoskeleton and soluble/insoluble material. The result obtained was intriguing since we observed a highly complex pattern of phosphorylation in insoluble material and in cytoskeleton; in parallel, we observed only one phosphorylated form of desmin in soluble fraction. These data suggest that phosphorylation, through a specific and complex pattern, could be involved in the organization of desmin IFs and not only in their disassembly. It is worth to note that a similar observation was previously done on vimentin, another type III intermediate filament protein. Indeed, it was observed that in contrast with phosphorylation by most other kinases, vimentin phosphorylation by MAPKAP kinase-2 seems to have no effect on its assembly^64^.

In parallel to phosphorylation, we observe a slight decrease of desmin O-GlcNAcylation following Thiamet G treatment, while O-GlcNAcylation and phosphorylation seems to be mutually exclusive on desmin. We have demonstrated that desmin is one of the substrates of OGT^30,44^, but the function of the glycosylation remains undefined. The O-GlcNAcylation of IFs was previously described on the type III IFs vimentin^7^, and occurs essentially in the head domain^6,33,65^. It was shown that O-GlcNAcylation of vimentin might block filament disassembly^33^ and is required for vimentin IF morphology and cell migration^6^. However, the only one site identified to date on desmin corresponds to Ser459^44^, i.e*.* in the tail domain, suggesting thus that O-GlcNAc could exert other functions than those expected. Of course, we cannot exclude that other O-GlcNAc site(s) may modify desmin; it should be interesting to go further in the characterization of the role of O-GlcNAcylation on desmin, in particular through the identification of additional modified sites.

Furthermore, the interplay between phosphorylation and O-GlcNAcylation was firstly mentioned in 1987^66^; since then, this interplay was extensively described on a large panel of proteins (for review, see^31,67–69^). Interestingly, it was also described that O-GlcNAcylation modulates kinome and phosphatome^70–72^. In addition, it was shown that OGT and OGA interact with kinases and phosphatases within multienzymatic complexes permitting the phosphorylation and/or O-GlcNAcylation of proteins of interest^33,39^. To explain the desmin phosphorylation change, we investigated whether or not kinases and phosphatases activity could be modified consecutively to OGT or OGA inhibition. The decrease of phosphorylation level could result from a decrease of kinase(s) activity and/or an increase in phosphatase(s) activity. Therefore, we applied a strategy without a priori to measure phosphatases activity^56,57^ and we didn’t quantify any changes in the activity of phosphatases. In parallel, we have selected the major kinases involved in desmin phosphorylation, i.e*.* PKA, PKC, PAK and CamKII^18^ and we determined that two kinases presented a variation of their activity and/or activation status. Thus, PKA was activated following the decrease of O-GlcNAc level (i.e*.* Ac_4_-5S-GlcNAc treatment); however, desmin phosphorylation remained unmodified. In contrast, the CamKII activation status decreased in Thiamet G-treated myotubes. This data suggest that the desmin phosphorylation decrease could result from a decrease of CamKII activity consecutively to global O-GlcNAcylation increase.

However, caffeine and the subsequent activation of CamKII by calcium didn’t modify phosphorylation of desmin. In contrast, we observed a decrease of desmin phosphorylation when CamKII was inhibited by KN-93, more specifically on the phosphoform that was modified after Thiamet G treatment. Interestingly, some data argue in favour of a modulation of the activation of CamKII through interactions with proteins partners such as α-actinin in a calcium-independent manner^73,74^. In this way, we previously observed that the interaction between α-actinin and protein partners was modulated consecutively to O-GlcNAcylation changes^47^. This data suggests that the modulation of CamKII activity could be much more intricate than those initially expected.

To sum, we have demonstrated in the herein paper that global O-GlcNAcylation increase led to a decrease of the desmin partition towards cytoskeleton correlated to a decrease of its phosphorylation that could result from a change of CamKII activity. Our data support the fact that the subcellular localization of desmin could be finely regulated by its PTMs signature in a spatio-temporal way involving O-GlcNAcylation. It should be thus essential to clarify the PTMs signature of desmin to fully understand the fine regulation of its functions and how this signature could be changed in the physiopathology of striated muscle disorders.

## Supplementary Information

Supplementary Information.

## Data Availability

The data and methods presented in the herein paper will be made available to other researchers upon reasonable request for purposes of reproducing the results or replicating the procedure.
