# Supplementary material for: Global O-GlcNAcylation changes impact desmin phosphorylation and its partition toward cytoskeleton in C2C12 skeletal muscle cells differentiated into myotubes
Source: Sci Rep. 2022 Jun 14;12:9831. doi: 10.1038/s41598-022-14033-z (PMC9198038; doi:10.1038/s41598-022-14033-z)

SUPPLEMENTAL DATA

Experimental procedures

- ***Cells viability assay***

Cells were cultured as described in the “Myoblasts proliferation and differentiation” paragraph, except that cells were platted in a 96-wells plate at a density of $1x10^5$ cells/ml. Following treatments, C2C12 myotubes were rinsed two-times with PBS at 37°C. Then, 100 µl of MTT ((3-(4,5-dimethylthiazol-2-yl)-2,5-diphenyltetrazolium bromide) diluted in PBS at a final concentration of 0,5 mg/ml were added for 3 hours at 37°C and saturated humidity. This colorimetric assay is based on the reduction of MTT, a yellow salt to purple formazan crystals in presence of metabolically active cells. Finally, MTT was removed and formazan crystals were solubilized in 100 µl of DMSO. The plate was read at 570 nm after agitation.

- ***Cells staining and differentiation***

The C2C12 cells were platted in 4-wells Lab-Tek chambers at a density of $2x10^5$ cells/ml. Treatments were applied on 5 days differentiated myotubes. Medium was then discarded and cells were rinsed two-times with PBS. Cells were fixed by methanol for 6 minutes with gentle agitation, rinsed three-times with PBS before being stain with May-Grünwald-Giemsa (Sigma-Aldrich). Myoblasts and myotubes were visualized using LEICA DMi8 microscope and images were acquired by LAS-X software. The differentiation was determined using the fusion index which was determined by the number of nuclei in myotubes (>2 nucleus) divided by the total number of nuclei.

- ***SDS-PAGE***

Denatured proteins in Laemmli Buffer were separated by SDS-PAGE on 7,5% or AnykD Stain-Free precast polyacrylamide gels (Mini-PROTEAN TGX Stain-free gels, Biorad). This electrophoretic separation was done at constant voltage (300V) for about 20 minutes in tank with migration buffer (190 mM Glycine; 25 mM Tris Base; 0,1% SDS); at the end of electrophoretic separation, total proteomes were visualized after UV activation with ChemiDoc MP Imager (Imaging System) and ImageLab software (Biorad) thanks to the Stain-Free technology. Then, proteins were transferred on 0,2 µm

nitrocellulose membrane using Trans-blot Turbo transfer system (Biorad) at 1,3 A, up to 25 V/gel for 10 minutes. The quality of transfer was performed by ChemiDoc Imager using the Stain-Free technology.

- ***Western-blot***

Then, membranes were blocked with 5% BSA for CamKII and P-CamKII antibodies or 5% non-fat milk for desmin, Akt, GAPDH, PAK1 and P-PAK1/2 antibodies in TBS-T (Tris Buffer Saline-Tween: 15 mM Tris/HCl, pH 7,6; 140 mM NaCl; 0.05% Tween-20). Membranes were incubated with primary antibody as follow: Desmin (Abcam ab6322) at 1/50000^{e}; GAPDH (Abcam ab9485) at 1/2000^{e}; Akt (Cell Signaling #92725) at 1/2000^{e}; PAK1 (Cell Signaling #2602) at 1/1000^{e}; P-PAK1/2 (Ser144/Ser141) (Cell Signaling #2606) at 1/1000^{e} in blocking solution overnight at 4°C with gentle agitation. After three washes of 10 minutes in TBS-T, membranes were incubated in blocking solution with secondary antibodies (HRP-linked IgG, Cell Signaling #7074 or #7076) during one hour at room temperature before washing five times during 10 minutes in TBS-T. Finally, ECL Clarity (Biorad) and ChemiDoc MP were used for chemiluminescence detection and image capture. Images were acquired and analysed using Image Lab® software.

<u>Supplemented figure 1</u>: **Cell viability and differentiation of 5-days differentiated C2C12 myotubes following Ac$_4$-5S-GlcNAc (5S, ▲) or Thiamet G (ThG, ■) treatment. (a)** Cell viability quantified using MTT colorimetric assay. **(b)** C2C12 myotubes differentiation using calculation of fusion index following May-Grünwald-Giemsa (MGG) staining. **(c-e)** Representative images of C2C12 of myoblasts and myotubes after MGG staining. Data were expressed as mean ± SEM and compared with the control condition.

(n = 8 for cell viability; n = 4 for cell differentiation).

<u>Supplemented figure 2:</u> **Protein profiles resulting from differential extraction of cytoskeletal proteins, and soluble/insoluble protein extraction.** Proteins were extracted from 5-days differentiated

myotubes. **(a)** Cytoskeleton extraction (left of the panel) permitted the obtainment of soluble proteins (S) and cytoskeletal proteins, while the second extraction protocol we used led to the soluble (S) and the insoluble (I) fraction (right of the panel). **(b-d)** All fractions were separated using SDS-PAGE, and expression of Akt (b), desmin (c) and GAPDH (d) was visualized between each fraction by western blot. Uncropped images of gel and blots are presented at the end of Supplemental data.

Supplemented figure 3: **Quantification without an *a priori* of phosphatase activities involved in desmin dephosphorylation.** Each signal, corresponding to a specific phosphatase activity, was separately quantified after in-gel detection of phosphatases. Uncropped image of gel is presented at the end of Supplemental data.

Supplemented figure 4: Determination of activation status of kinases involved in desmin phosphorylation. Representative images of western blots performed to determine the phosphorylation level of CamKII and PAK and their whole protein level, the phosphorylation level translating their activation status. **(a)** Phosphorylation status of CamKII. **(b)** Phosphorylation status of PAK. Uncropped images of gels and blots are presented at the end of Supplemental data.

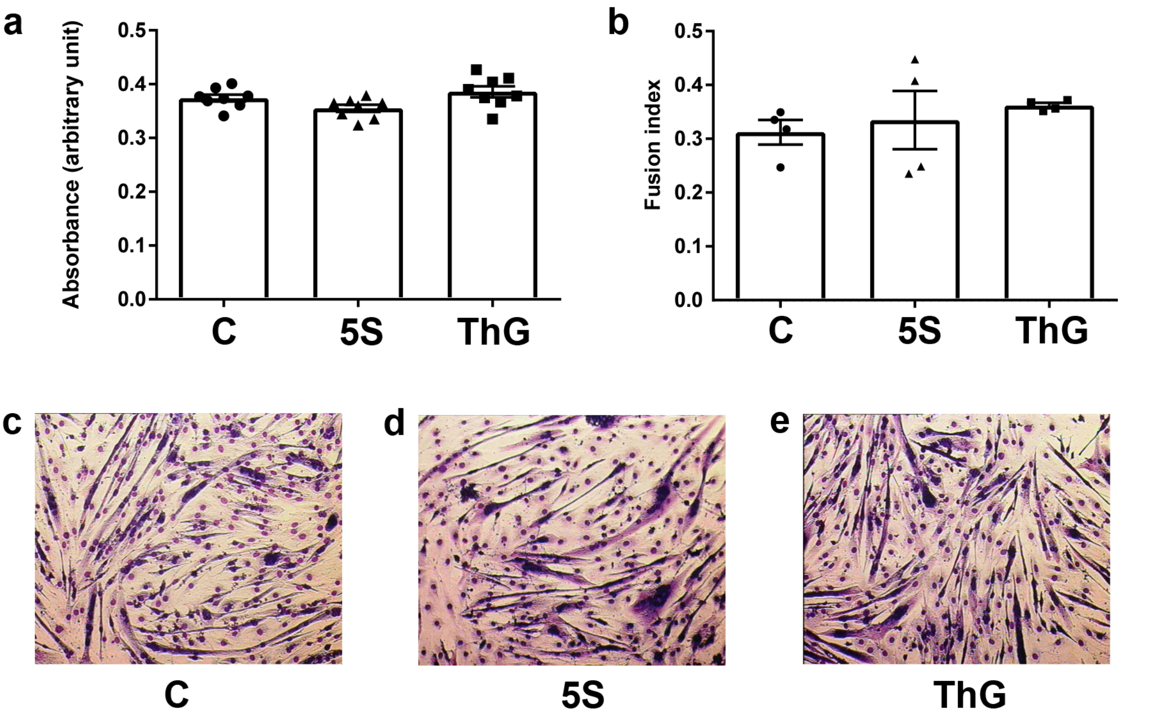

Supplemented Figure 1

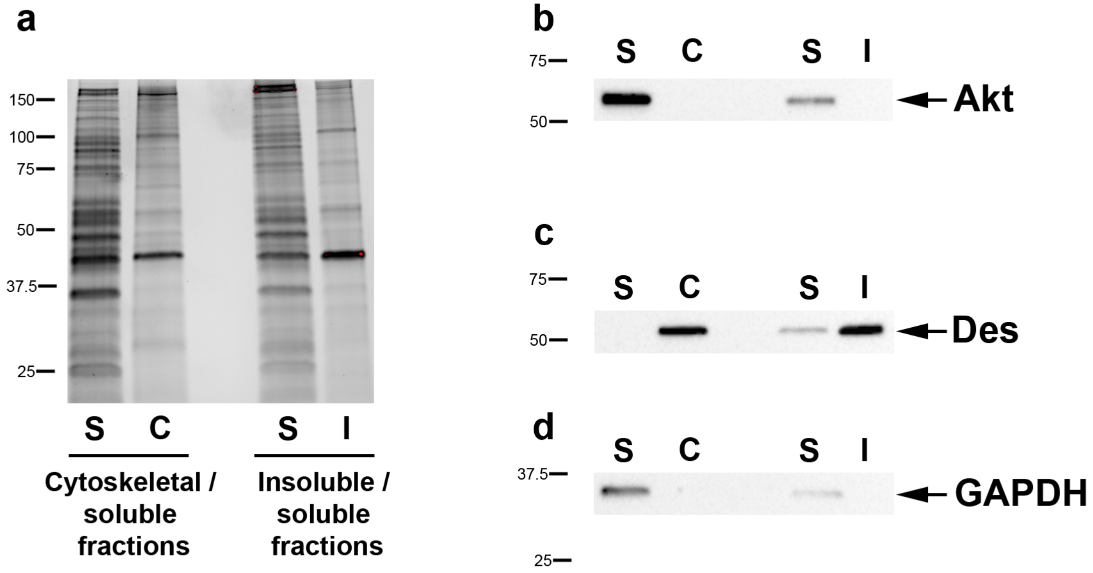

Supplemented Figure 2

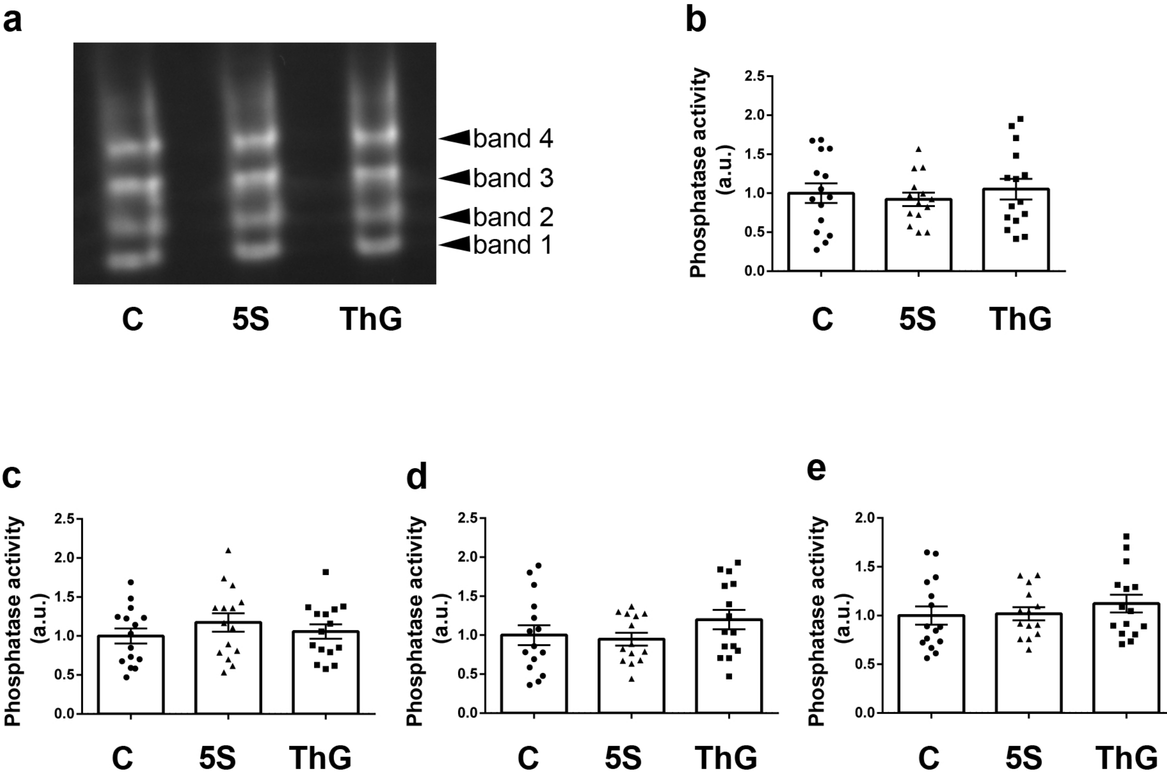

Supplemented Figure 3

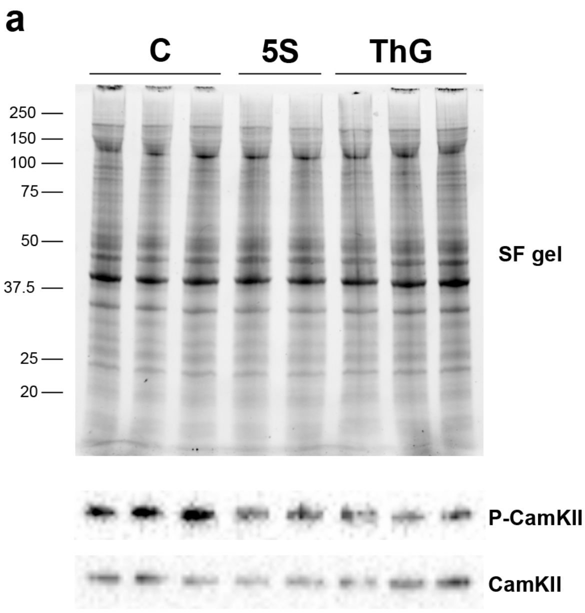

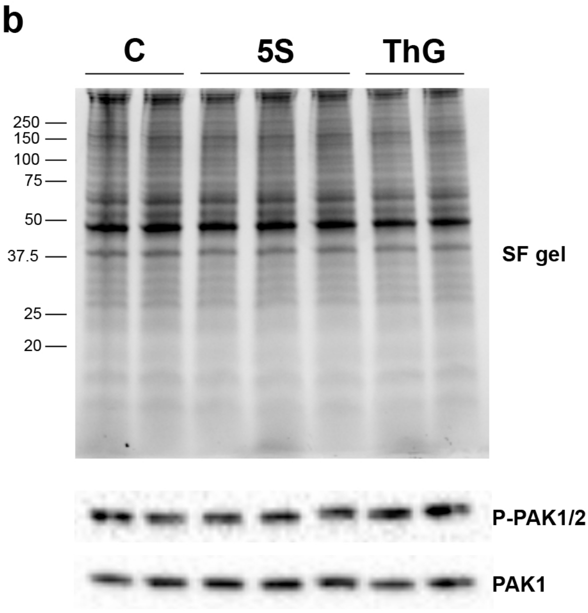

Supplemented Figure 4

Uncropped images for RL-2 Western blot for Figure 2

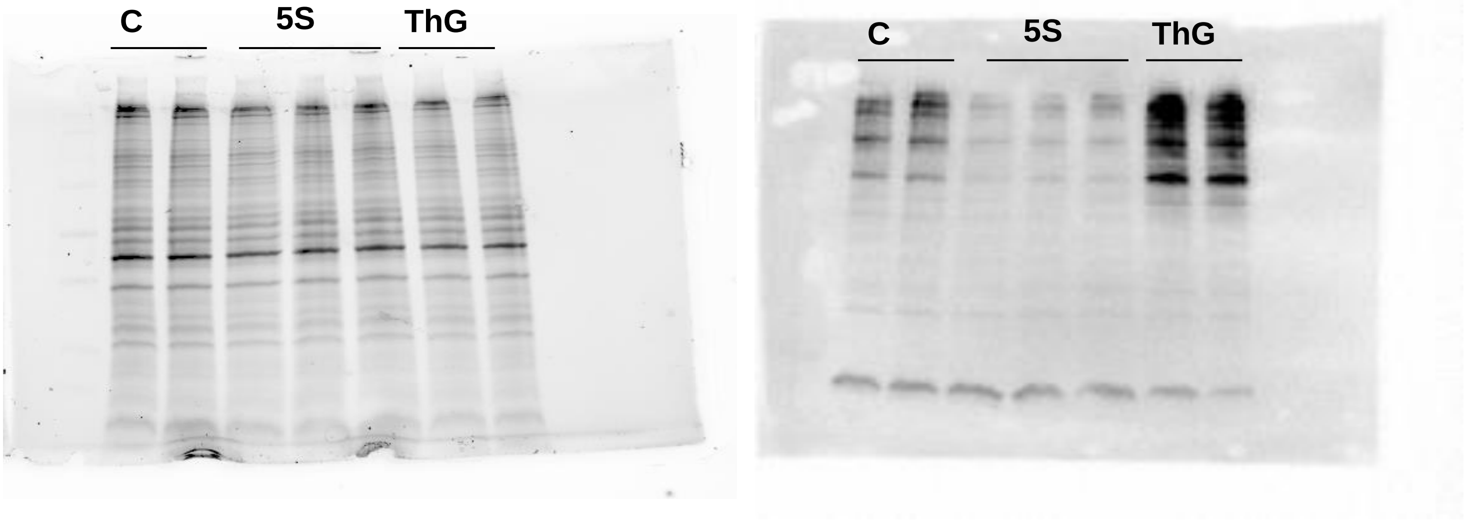

Uncropped images for desmin Western blot after PhosTag-PAGE for Figure 4a

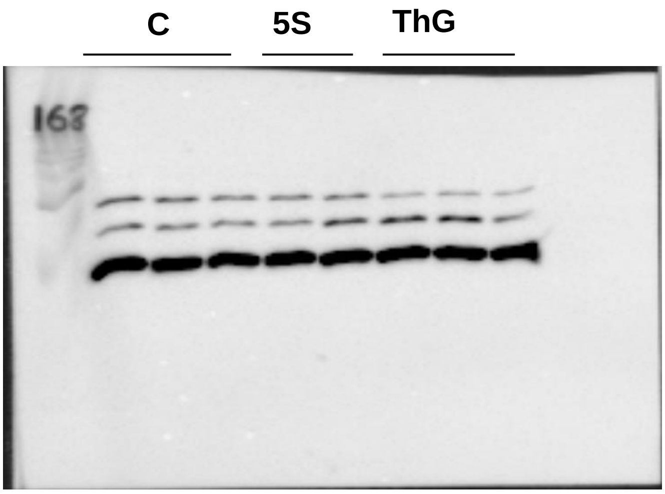

Uncropped images for desmin Western blot after PhosTag-PAGE for Figure 4c

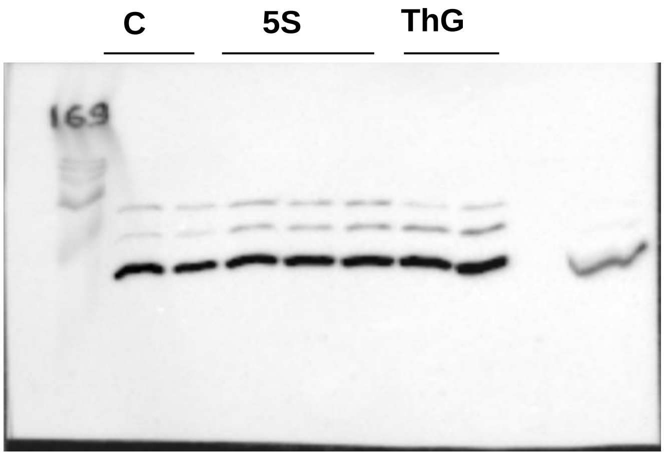

Uncropped images for desmin Western blot after PhosTag-PAGE in Figure 4d

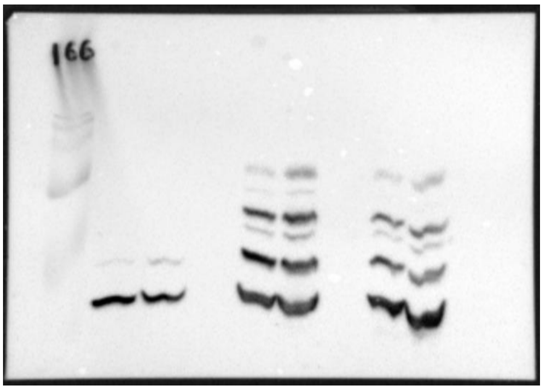

Uncropped images for desmin Western blot for Figure 5a (WE : whole extract; IP : immunoprecipitation)

WE

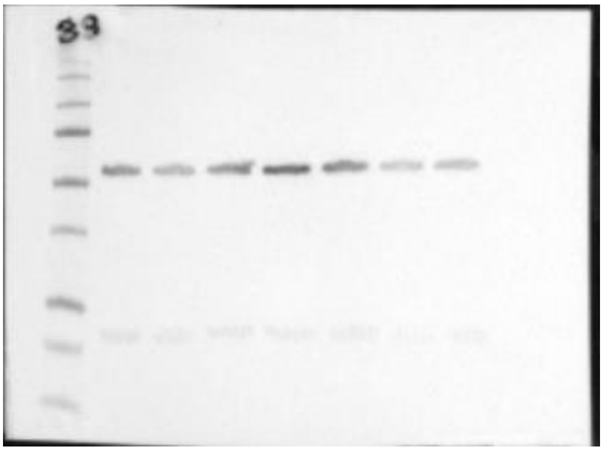

IP

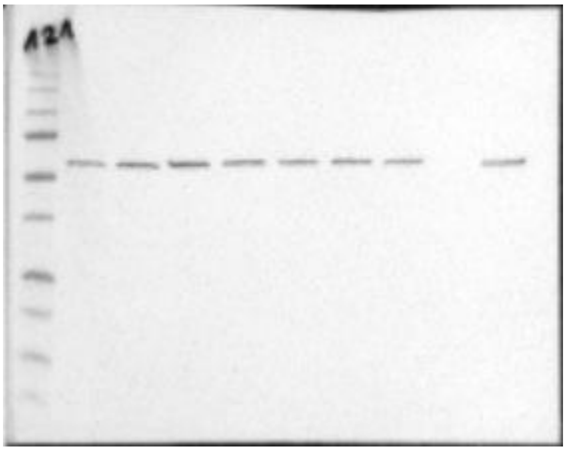

Uncropped images for desmin Western blot after RL-2 immunoprecipitation and PhosTag-PAGE for Figure 5b

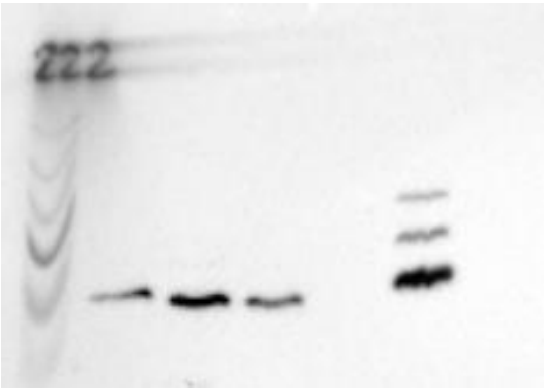

Uncropped images for MUP gels for Figure 6 and Supplemented Figure 3a

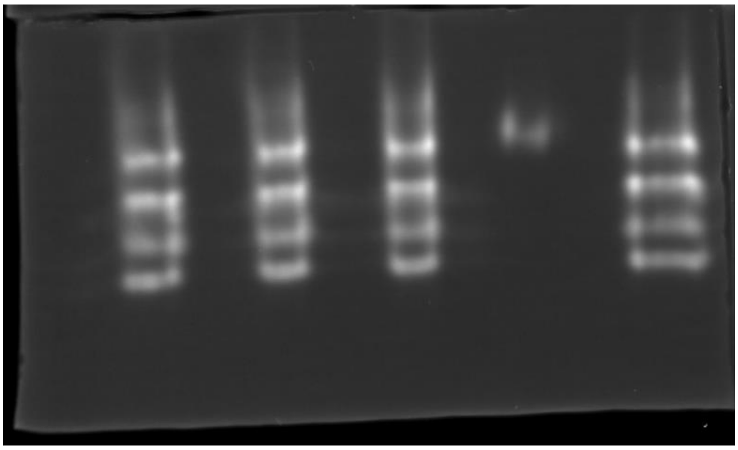

Uncropped images for CamKII western blot for Figure 8

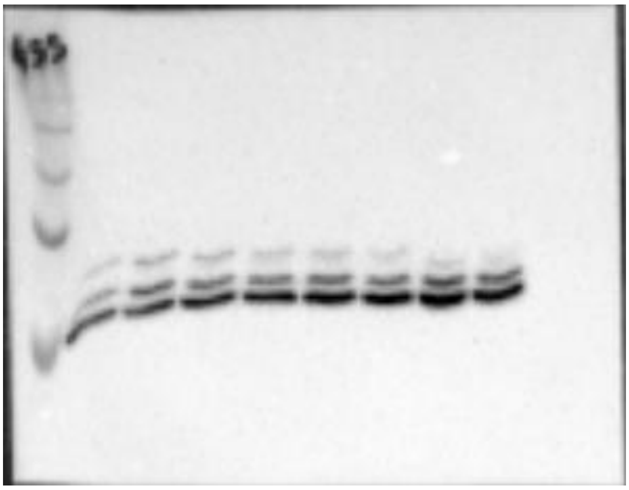

Uncropped images for Stain free gel and Akt, desmin and GAPDH western blot for Supplemented Figure 2

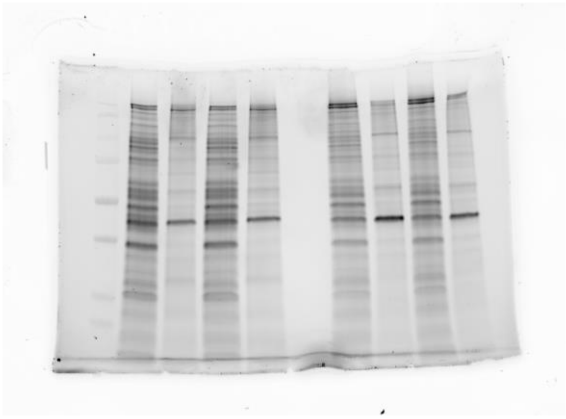

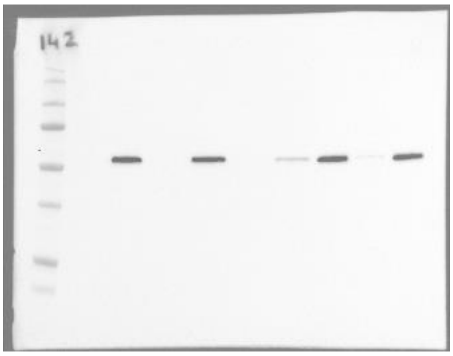

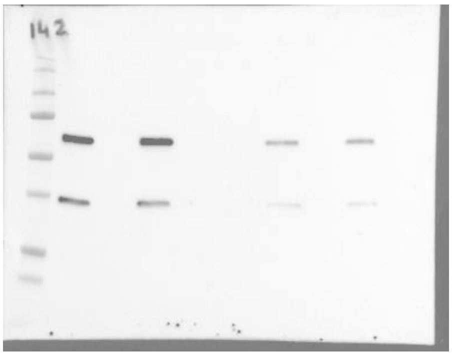

Uncropped images for Stain free gel and P-CamKII and CamKII for Supplemented Figure 4a

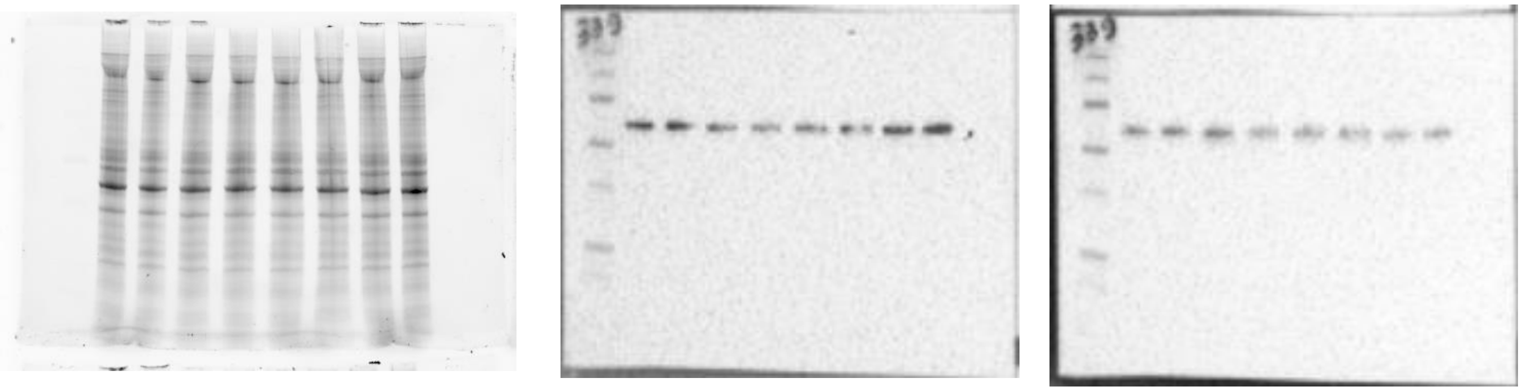

Uncropped images for Stain free gel and P-PAK1/2 and PAK1 for Supplemented Figure 4b

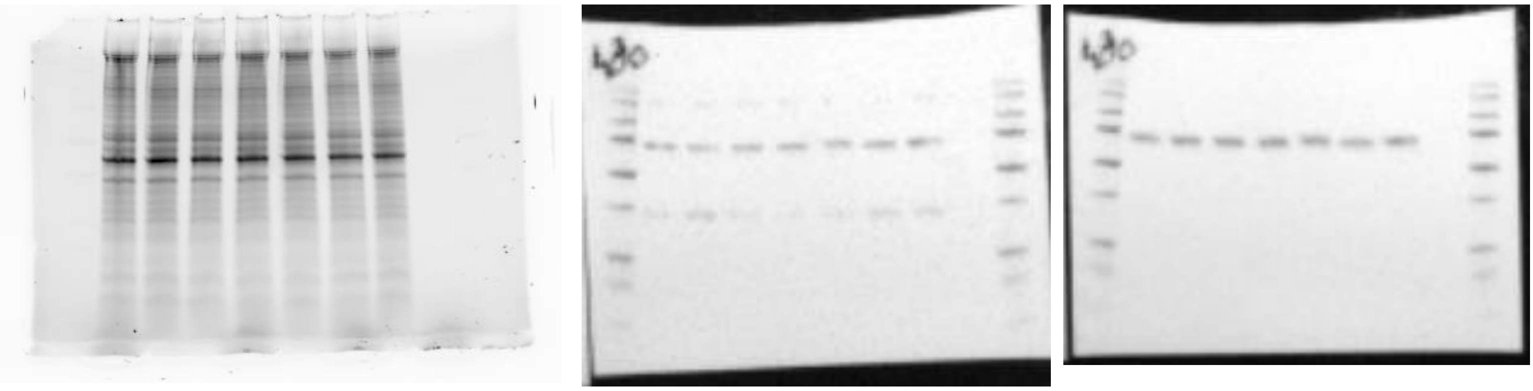

Supplement: Supplementary file 1 — Supplementary Information. [file 41598_2022_14033_MOESM1_ESM.pdf]
